# Differences in Stroke or Systemic Thromboembolism Readmission Risk After Hospitalization for Atrial Fibrillation and Atrial Flutter

**DOI:** 10.7759/cureus.23844

**Published:** 2022-04-05

**Authors:** Harshith S Thyagaturu, Alexander Bolton, Sittinun Thangjui, Kashyap Shah, Bishesh Shrestha, Dinesh Voruganti, Daniel Katz

**Affiliations:** 1 Internal Medicine, Bassett Medical Center, Cooperstown, USA; 2 Hospitalist, UnityPoint Health - St. Luke’s Hospital, Cedar Rapids, USA; 3 College of Public Health, University of Iowa, Iowa City, USA; 4 Internal Medicine, St. Luke’s University Health Network, Bethlehem, USA; 5 Cardiology, Bassett Medical Center, Cooperstown, USA; 6 Cardiology, University of Arkansas for Medical Sciences, Little Rock, USA

**Keywords:** readmission, systemic thromboembolism, stroke, atrial flutter, atrial fibrillation

## Abstract

Background

Although atrial fibrillation (AF) and atrial flutter (AFL) are different arrhythmias, they are assumed to confer the same risk of stroke and systemic thromboembolism (STE) despite a lack of available evidence. In this study, we investigated the difference in the risk of stroke or STE after AF and AFL hospitalizations.

Methodology

The National Readmission Database (NRD) 2018 was used to identify AF and AFL patients using appropriate International Classification of Diseases, Tenth Revision, Clinical Modification (ICD-10-CM) codes and were followed until the end of the calendar year to identify stroke or STE readmissions. Survival estimates were calculated, and a Cox proportional hazards model was used to calculate the adjusted hazards ratio (aHR) and compare the risk of stroke or STE readmissions between AF and AFL groups.

Results

A total of 215,810 AF and 15,292 AFL patients were identified. AFL patients were more likely to be younger (66 vs. 70 years), male (68% vs. 47%), and had higher prevalence of obesity (25% vs. 22%), obstructive sleep apnea (14% vs. 12%), diabetes mellitus (31% vs. 26%), and alcohol use (6.9% vs. 5.5%) (all p < 0.01). After adjusting for potential patient and hospital-level characteristics, there was a statistically significant decrease in one-year stroke or STE readmission risk in AFL patients compared to AF patients (aHR 0.79 (0.66-0.95); p = 0.01).

Conclusions

AFL patients are commonly younger males with a higher burden of medical comorbidity. There is a decrease in the one-year risk of stroke or STE events in AFL patients compared to AF. The predictors of stroke and STE are similar in both AFL and AF groups. Further studies with longer follow-up and anticoagulation data are needed to verify the results.

## Introduction

Atrial fibrillation (AF) is associated with an increased risk of cardioembolic strokes and systemic thromboembolism (STE) [1-3]. Despite the common impression that AF and atrial flutter (AFL) possess a similar stroke or STE risk, the relationship between AFL and stroke/STE has been addressed only in a few studies [4,5]. Furthermore, CHA2DS2-VASc scoring has not been well established for AFL patients [6]. Although AF and AFL are distinct arrhythmias, they tend to co-exist within patients [7]. The formation of STE in AF is evidenced to be multifactorial, with one of the reasons being abnormal blood flow leading to stasis in the left atrium and left atrial appendage (LAA) [8]. Studies show a lower risk of LAA clot formation and, theoretically, a lower risk of cardioembolic stroke and STE in AFL [9]. However, the currently available evidence on this topic is inconclusive, with uncertainty in the long-term thromboembolic risk difference between AFL and AF. Therefore, the American College of Cardiology/American Heart Association/Heart Rhythm Society’s 2019 focused update recommends for AFL patients the same AF stroke risk assessment and anticoagulation strategies [10]. However, in the 2019 European Society of Cardiology’s guidelines for managing supraventricular tachycardia, the threshold for anticoagulation initiation in AFL patients without AF was not established [11]. Using the National Readmission Database (NRD), we investigated the difference in the risk of stroke or STE readmissions between AFL and AF. In addition, we investigated the predictors of stroke and STE rehospitalizations in both groups.

## Materials and methods

Data source

We conducted a retrospective cohort study using the 2018 NRD. The NRD is a database developed for the Healthcare Cost and Utilization Project (HCUP) sponsored by the Agency for Healthcare Research and Quality through a Federal-State-Industry partnership. In 2018, the NRD contained data from 28 geographically dispersed states accounting for approximately 60% of the total US resident population and 58.7% of all US hospitalizations [12]. It contains reliable, verifiable patient linkage numbers (defined as the “NRD_VISITLINK” variable within the dataset) that can track a patient across hospitals within the same state while adhering to strict privacy guidelines. The NRD comprises more than 100 clinical and non-clinical variables for each hospital stay. Each discharge is weighted to calculate national estimates. The NRD or administrative data have been previously used to provide reliable national stroke risk estimates through readmissions [13,14]. The NRD in the year 2018 contained patient and hospital-level data with up to 40 diagnoses and 25 procedures for each patient using appropriate International Classification of Diseases, Tenth Revision, Clinical Modification (ICD-10-CM) codes. Institutional Review Board approval was not required due to the deidentified nature of the data. We followed the checklist that has been recommended by HCUP for working with the NRD [15].

Study population and outcome

**Figure 1:**
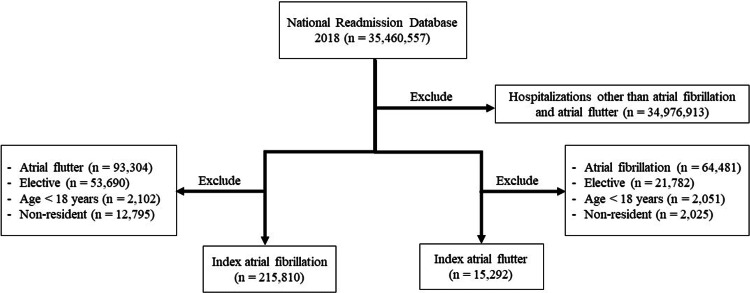
Inclusion diagram: flow diagram describing the inclusion and exclusion criteria and their application to the population within the National Readmission Database, 2018.

Index AF and AFL admissions were identified by the presence of their respective ICD-10-CM codes as the primary diagnosis for that hospitalization (see Table 1 for the list of ICD-10-CM codes used in this study). Patients with secondary AFL diagnosis in the AF index admission group and patients with secondary AF diagnosis in the AFL index admission group were excluded to mitigate the overlap of AF and AFL within the same patient. We excluded patients with age ≤18 years, trauma-related readmissions, and elective readmissions. Further details regarding inclusion and exclusion criteria are shown in Figure 1.

**Table 1:** ICD-10-CM codes used in this study. ICD-10-CM: International Classification of Diseases, Tenth Revision, Clinical Modification

Diagnosis	ICD10-CM codes
Atrial fibrillation	I48.0, I48.1, I48.2, I48.91
Atrial flutter	I48.3, I48.4, I48.92
Stroke	G45, G46, I63, I64, I67.81, I68.782, I69.8, I69.3, H34.1
Systemic thromboembolism	I74, K55.0, H34.0, H34.2
Congestive heart failure	I50
Hypertension	I10, I11, I12, I13, I14, I15, I16
Diabetes mellitus	E08, E09, E10, E11, E13
History of stroke	I69.3, Z86.73
Peripheral vascular disease	E08.5, E09.5, E10.5, E11.5, E13.5, I73, T82.856, Z98.62, Z95.820, I25.2, I25.83
Hyperthyroidism	E03
Obstructive sleep apnea	G47.33
Coronary artery disease	I20, I21, I22, I23, I24, I25
History of coronary artery bypass grafting	I25.70, I25.71, I25.72, I25.73, I25.76, I25.79, I25.810, T82.21, Z95.1
Chronic obstructive pulmonary disease	J41, J42, J43, J44
Obesity	E66, Z683, Z684
Chronic kidney disease ≥Stage 3	N183, N184, N185, E082, E132, I12, I13
End-stage renal disease	N186, Z992, Z4931, Z4901
Anemia	D50, D51, D52, D53, D55, D56, D57, D58, D59, D60, D61, D62, D63, D64, D46.0, D46.1, D46.2, D46.4, O99.0
Smoking	F17, Z87.891
Alcohol	F10

The first observed AF and AFL hospitalization in the NRD 2018 was defined as the index admission. These patients were followed until the end of the 2018 academic year. The primary outcome of our study was to identify and compare ischemic stroke and STE readmission rates after AF and AFL index admissions. The stroke or STE readmissions were identified by their ICD-10-CM codes (see Table 1) in the primary or secondary diagnosis sections of readmissions.

Statistical analysis

Statistical analyses were performed using the Stata software package, version 17.0 (StataCorp, College Station, TX). Stata’s survey package facilitates analysis by considering NRD’s complex sampling design that includes stratification, clustering, and weighting to produce national estimates. We used the chi-square test to evaluate the differences between groups of categorical variables and the Student’s t-test for differences between sample means of continuous variables. Survival analysis was performed with time from index hospitalization discharge to readmission as the time variable and stroke or STE readmissions as the failure variable to produce Kaplan-Meier curves. Patients who did not experience failures were censored on day 365 after discharge. Univariate Cox regression analysis was performed to calculate the unadjusted hazard ratio (HR) for the primary outcome. Subsequently, multivariate Cox regression analysis was used to adjust for potential confounders and produce adjusted hazard ratios (aHRs). Multiple covariates were built into the model based on the clinical experience of the authors, currently available literature, and significantly associated with the outcome on univariate analysis with a p-value < 0.2. All p-values were calculated based on two-tailed tests, with 0.05 as a threshold for statistical significance.

## Results

Baseline characteristics of index admissions

We identified 215,810 weighted index admissions with AF and 15,292 with AFL (See Table 2 for demographic data). The AFL group was younger than the AF group (mean age of 66.7 years vs. 70.1 years; p < 0.01), with approximately 70% of patients <75 years of age, and they were more commonly associated with males. There was no difference in the mean Charlson comorbidity score between the AFL and AF groups, but the AFL group was associated with a lower CHA2DS2-VASc score compared to the AF group (mean score of 2.8 vs. 3.3; p < 0.01). Hypertension, diastolic heart failure, a history of stroke, and hyperthyroidism were more commonly associated with the AF group, whereas obesity, obstructive sleep apnea, diabetes mellitus, and nicotine dependence were associated with the AFL group.

**Table 2:** Baseline characteristics of the atrial fibrillation and atrial flutter groups. *ICD-10-CM codes were utilized to identify the comorbidities, which are reported in Table 1. CAD = coronary artery disease; CHF = congestive heart failure; CKD = chronic kidney disease; COPD = chronic obstructive pulmonary disease; ESRD = end-stage renal disease; ICD-10-CM = International Classification of Diseases, Tenth Revision, Clinical Modification; OSA = obstructive sleep apnea

Variables	Atrial fibrillation (n = 215,810)	Atrial flutter (n = 15,292)	P-value
Age (years) (mean)	70.1	66.7	<0.01
18–49	7.2 (%)	9.2 (%)	
50–64	23.0 (%)	33.0 (%)	
65–74 (%)	26.1 (%)	28.4 (%)	
>75 (%)	43.7 (%)	29.3 (%)	
Indicator of gender			<0.01
Male	47.4 (%)	68.1 (%)	
Female	52.6 (%)	32.0 (%)	
Charlson Comorbidity Index Score			<0.01
0	25.9 (%)	26.3 (%)	
1	27.7 (%)	26.6 (%)	
2	19.3 (%)	19.9 (%)	
3	11.4 (%)	11.2 (%)	
≥4	15.6 (%)	15.9 (%)	
Mean Charlson Comorbidity Score	1.8	1.8	0.38
CHA_2_DS_2_-VASc score			<0.01
0	4.4 (%)	6.8 (%)	
1	11.0 (%)	15.3 (%)	
2	16.4 (%)	23.2 (%)	
3	20.5 (%)	22.6 (%)	
4	22.5 (%)	17.5 (%)	
≥5	25.1 (%)	14.4 (%)	
Mean CHA_2_DS_2_ VASc score	3.3	2.8	<0.01
Comorbidities*			
Obesity	22.4 (%)	25.5 (%)	0.03
OSA	12.7 (%)	14.6 (%)	<0.01
Hypertension	78.5 (%)	72.8 (%)	<0.01
Diabetes mellitus	26.2 (%)	31.3 (%)	<0.01
COPD	16.4 (%)	15.7 (%)	0.14
CAD	33.6 (%)	33.4 (%)	0.29
Heart failure	36.0 (%)	32.9 (%)	<0.01
Systolic heart failure	16.3 (%)	18.6 (%)	0.35
Diastolic heart failure	14.2 (%)	10.4 (%)	<0.01
Prior stroke	8.3 (%)	6.0 (%)	<0.01
CKD stage ≥3	14.2 (%)	14.1 (%)	0.85
ESRD	1.5 (%)	2.0 (%)	<0.01
Peripheral vascular disease	11.2 (%)	11.2 (%)	0.97
Anemia	12.8 (%)	11.2 (%)	<0.01
Hyperthyroidism	17.0 (%)	12.4 (%)	<0.01
Smoker	37.4 (%)	43.5 (%)	<0.01
Alcohol	5.5 (%)	6.9 (%)	<0.01

Difference in the risk of stroke or STE readmissions

**Figure 2:**
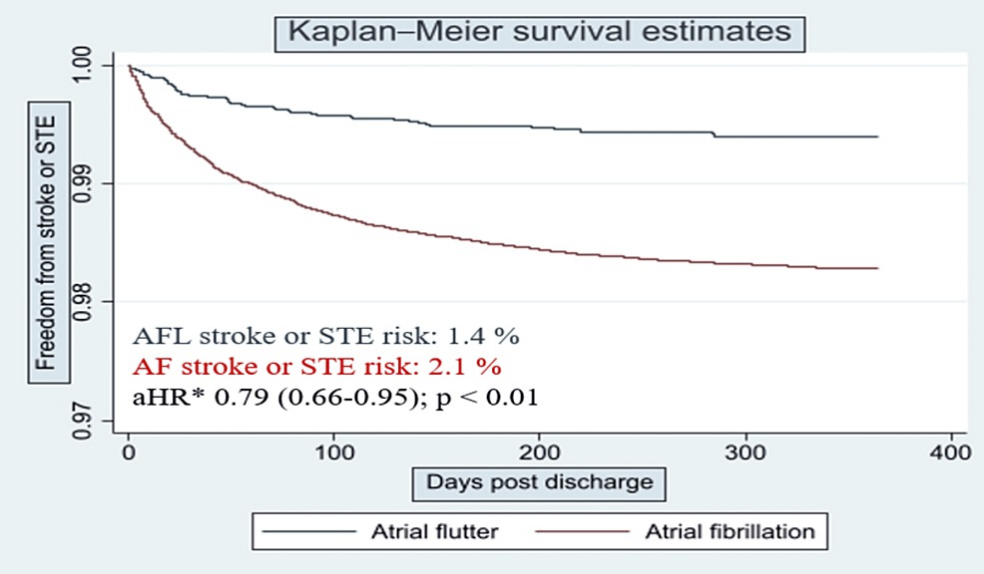
Kaplan-Meier survival estimates showing the differences in the risk of stroke and systemic thromboembolism readmissions between atrial fibrillation and atrial flutter patients, National Readmission Database, 2018. The atrial flutter (blue) group was significantly associated (p < 0.01) with a lower risk of stroke or systemic thromboembolism readmissions compared to the atrial fibrillation group. *Adjusted hazards for atrial flutter compared to the atrial fibrillation group. aHR = adjusted hazards ratio; STE = systemic thromboembolism

After adjusting for potential confounders (age, gender, hypertension, diabetes, history of prior stroke, chronic kidney disease (CKD), obesity, obstructive sleep apnea, Charlson comorbidity score, CHADS VASc score, malignancy, smoking, and alcohol status), AFL was associated with lower hazards of stroke or STE readmissions for a year (1.4% for AFL vs. 2.1% for AF; aHR = 0.79 (0.66-0.95); p < 0.01) (Table 3). Figure 2 shows the Kaplan-Meier survival curves comparing the AF and AFL groups for the primary outcome.

**Table 3:** Risk of stroke or systemic thromboembolism readmissions within a year in atrial fibrillation and atrial flutter patients. *Hazards of stroke or STE in atrial flutter patients compared to atrial fibrillation patients. HR = hazard ratio; STE = systemic thromboembolism

	Atrial fibrillation	Atrial flutter	Unadjusted hazard ratio*	P-value	Adjusted hazard ratio*	P-value
Stroke or STE readmissions	4,675 (2.1%)	226 (1.4%)	0.67 (0.56–0.81)	<0.01	0.79 (0.66–0.95)	0.01

Predictors of stroke or STE readmissions after AF and AFL

Age, female gender, higher Charlson comorbidity score, higher CHA2DS2 VASc score, hypertension, diabetes, coronary artery disease, history of stroke, CKD stage ≥3, and peripheral vascular disease were identified as independent predictors of stroke or STE in the AFL patients. Similar factors were identified in AF patients. However, hypertension, diabetes mellitus, and peripheral vascular disease status showed similar risk and CKD stage ≥3 showed a decreased risk of stroke or STE in the AF group (see Table 4 and Table 5).

**Table 4:** Predictors of stroke or systemic thromboembolism readmissions after index atrial fibrillation admission, National Readmissions Database, 2018. *Variables used for calculating adjusted hazard ratio: age, female, Charlson comorbidity index, CHA2DS2 VASc score, hypertension, diabetes mellitus, obesity, congestive heart failure, prior CABG, obstructive sleep apnea, prior stroke, COPD, CKD ≥3, ESRD, peripheral vascular disease, anemia, alcohol use disorder. The adjusted hazard ratio depicts the risk for stroke or systemic thromboembolism readmissions with atrial fibrillation admissions. CABG = coronary artery bypass graft; COPD = chronic obstructive pulmonary disease; CKD = chronic kidney disease

Variables	aHR*	95% CI	P-value
Age	1.01	1.01–1.02	0.01
18–49	Reference		
50–64	2.18	1.62–2.93	<0.01
65–74	3.50	2.63–4.66	<0.01
>75	4.95	3.74–6.54	<0.01
Gender			
Male	Reference		
Female	1.10	0.99–1.22	0.05
Charlson Comorbidity Index Score			
0	Reference		
1	1.31	1.12–1.53	<0.01
2	1.65	1.40–1.94	<0.01
3	2.21	1.83–2.66	<0.01
≥4	3.16	2.60–3.83	<0.01
CHA_2_DS_2_ VASc score			
0	Reference		
1	2.18	1.15–4.11	0.01
2	2.54	1.36–4.74	<0.01
3	3.64	1.92–6.91	<0.01
4	4.42	2.29–8.52	<0.01
≥5	4.16	2.06–3.87	<0.01
Comorbidities			
Hypertension	1.14	0.98–1.33	0.07
Diabetes mellitus	0.92	0.82–1.02	0.14
Obesity	0.81	0.72–0.91	<0.01
Coronary artery disease	1.17	1.06–1.29	<0.01
Congestive heart failure	0.96	0.87–1.07	0.54
Prior CABG	1.14	0.98–1.32	0.08
Obstructive sleep apnea	0.87	0.75–1.00	0.06
COPD	1.04	0.92–1.14	0.44
Prior stroke	2.17	1.91–2.00	<0.01
Chronic kidney disease stage ≥3	0.78	0.68–0.89	<0.01
End-stage renal disease	1.55	1.19–2.00	<0.01
Peripheral vascular disease	1.05	0.92–1.20	0.44
Anemia	1.04	0.93–1.16	0.41
Nicotine dependence	0.95	0.87–1.04	0.23
Alcohol use disorder	1.14	0.92–1.40	0.20

**Table 5:** Predictors of stroke or systemic thromboembolism readmissions after index atrial flutter admissions, National Readmissions Database, 2018. *Variables used for calculating adjusted hazard ratio: age, female, Charlson comorbidity index, CHA2DS2 VASc score, hypertension, diabetes mellitus, obesity, congestive heart failure, prior CABG, obstructive sleep apnea, prior stroke, COPD, chronic kidney disease stage ≥3, end-stage renal disease, peripheral vascular disease, anemia, alcohol use disorder. The adjusted hazard ratio depicts the risk for stroke or systemic thromboembolism after atrial flutter admissions. CABG = coronary artery bypass graft; COPD = chronic obstructive pulmonary disease

Variables	aHR*	95% CI	P-value
Age	1.01	1.01–1.02	<0.01
18–49	Reference		
50–64	2.78	1.00–7.74	0.05
65–74	2.59	0.91–7.31	0.07
>75	5.07	1.85–13.9	<0.01
Gender			
Male	Reference		
Female	1.62	1.14–2.31	<0.01
Charlson Comorbidity Index Score			
0	Reference		
1	1.63	0.84–3.13	0.14
2	2.31	1.20–4.43	0.01
3	2.10	1.01–4.37	0.04
≥4	4.20	2.30–7.65	<0.01
CHA_2_DS_2_ VASc score			
0	Reference		
1	0.75	0.17–3.19	0.70
2	2.04	0.59–6.99	0.25
3	3.16	0.95–10.5	0.06
4	4.36	1.32–14.3	0.01
≥5	6.63	2.02–21.7	<0.01
Comorbidities			
Hypertension	1.92	1.17–3.15	<0.01
Diabetes mellitus	1.59	1.12–2.27	<0.01
Obesity	0.81	0.51–1.28	0.37
Coronary artery disease	1.73	1.22–2.47	<0.01
Congestive heart failure	1.09	0.75–1.58	0.65
Prior CABG	1.81	1.09–1.99	0.02
Obstructive sleep apnea	0.86	0.49–1.52	0.62
COPD	1.42	0.94–2.15	0.09
Prior stroke	3.39	2.06–5.58	<0.01
Chronic kidney disease stage ≥3	1.88	1.24–2.87	<0.01
End-stage renal disease	2.14	0.93–4.93	0.07
Peripheral vascular disease	2.75	1.84–4.13	<0.01
Anemia	1.55	0.97–2.47	0.06
Nicotine dependence	1.25	0.87–1.80	0.21
Alcohol use disorder	1.08	0.51–2.28	0.84

## Discussion

Our study demonstrates evidence of a reduced risk of readmission for stroke or STE in AFL patients compared to AF patients. On average, AFL patients were younger, more likely to be male, and have a lower mean CHA2DS2-VASc score. However, the AFL cohort had a higher burden of relevant comorbidities such as diabetes, end-stage renal disease, and obesity, as well as tobacco and alcohol use. Yet, after adjustment for these covariates in our final model, the AFL cohort maintained a 20% reduced risk of stroke or STE readmission compared to the AF cohort.

While AFL is electrophysiologically distinct from AF, epidemiologic studies describing AFL alone are limited. Data from the Framingham Heart Study on 112 patients with AFL who were matched based on age and sex with AF patients as well as healthy controls revealed that, compared to controls, patients who smoked, had moderate-to-heavy alcohol use, and history of myocardial infarction and/or heart failure were associated with AFL incidence. Compared to AF, AFL patients had less heart valve disease [16]. Our cohort of hospitalized patients with AFL also had significant levels of alcohol and tobacco use disorders, and compared to AF patients, alcohol and tobacco use disorders were more prevalent in AFL patients. While a trend was seen toward more alcohol and tobacco use in AFL within the Framingham Heart Study, it was not significant at the 95% level (smoking: adjusted odds ratio (aOR) = 1.47, 95% confidence interval (CI) = 0.79-2.72; moderate-to-heavy alcohol use: aOR = 1.58, 95% CI = 0.68-3.68) [16]. We were likely able to identify a significant difference between AFL and AF for these disorders due to the greater size of our AFL cohort. Previous studies using animal models have demonstrated how tobacco [17,18] and alcohol [19] are associated with atrial arrhythmia formation, and while specific literature describing the relationship between tobacco and AFL is limited, alcohol has been associated with AFL formation in humans [20,21]. Further studies would be beneficial to examine the specific risks of alcohol, tobacco, and other potential risk factors for AFL, ideally with large enough cohorts to detect modest but significant differences.

The findings of our study are an addition to the limited evidence base evaluating the risk of stroke in AFL. Previous studies reported similar rates of stroke and/or STE risk in small groups of AFL patients, without any comparison to an AF patient group. Wood et al. reported an annual stroke risk of 1.6% in their 86 AFL patients referred for radiofrequency ablation with a mean follow-up of 4.5 years [5]. Another study with similar findings was reported by Seidl et al., with an annual risk of approximately 1.8% for thromboembolic events in their 191 AFL patients [4].

A few other studies evaluated the risk of AFL in comparison to a cohort of AF patients. Rahman et al. used data from the Framingham Heart Study to determine 10-year stroke outcomes in 96 AFL and 359 AF patients. There was no difference in stroke risk between AFL and AF, with approximately 1.2% annual risk in both groups [16]. Halligan et al. reported that, in their group of 59 patients with lone AFL, 19 (32%) experienced a cerebrovascular event (defined as either a transient ischemic attack or a stroke). When compared to a cohort of 145 patients with AF and after adjustment for age and sex, the AFL cohort had a higher incidence of a cerebrovascular event (aHR = 2.6, 95% CI = 1.2-5.3, p-value 0.011) [22]. However, it was notable that in the study by Halligan et al., six of the 19 AFL patients who had a cerebrovascular event (32%) developed AF after their AFL diagnosis, but prior to the cerebrovascular event, raising questions as to whether AFL or AF was the predominant arrhythmia causing the event. A similar issue occurred in a study by Al-Kawaz et al. who performed a large retrospective cohort study using administrative claims data from 2008 to 2014 on a 5% sample of Medicare beneficiaries. The study demonstrated a decreased annual incidence of stroke in AFL (1.4%) compared to AF patients (2.0%) [23]. However, there was also a significant conversion rate from AFL to AF (66%) across a mean follow-up time of 2.8 years.

A common limitation of research into stroke and STE events in AFL is that similar to the studies by Halligan et al. [22] and Al-Kawaz et al. [23], AF eventually develops in many patients who initially present with AFL [24], and efforts to isolate a lone AFL cohort that does not develop AF are difficult, especially considering how AF can present asymptomatically [25]. We attempted to limit this issue within our study by excluding patients who had codes for both AF and AFL at index hospitalization. However, a limitation remains in our study that we cannot fully exclude the possibility that some patients with AFL in our study also have AF, and vice-versa, due to the potential for inaccurate coding within the dataset, thus raising the potential for misattribution bias. Future research will ideally develop methods of ensuring as little crossover as possible between AFL and AF groups to determine the true stroke and STE risk of AFL more definitively compared to AF.

For our cohort of AFL, increased age and female sex, as well as hypertension, diabetes, previous stroke, and peripheral vascular disease were found to be significant predictors of stroke and STE readmission (see Table 4); coincidentally, these are many of the same predictors of stroke that exist for AF as well [26]. According to the 2014 AHA/ACC/HRS guidelines on the management of AF, it is a Class I recommendation to manage the risk of stroke similar to AF, as far as using a CHA2DS2-VASc score to guide decision-making regarding anticoagulation [27]. However, this recommendation was made based on expert opinion, without citation of evidence. While our research supports this decision by identifying many of the same risk factors for stroke in AFL as for AF, future research into whether patients with AFL would benefit from different risk factor assessments for stroke compared to AF patients would be beneficial to establish an evidence basis for clinical decision-making.

Limitations

Our study has several limitations that are common in studies of administrative data. As stated previously, our data are derived from administrative codes that are used for insurance billing rather than clinical purposes. As such, they are subject to misclassification bias from inaccurate entries or missing codes [28]. However, the missing data among the variables we used was less than 2% in total and unlikely to cause significant changes to our results. In addition, we used diagnosis codes for AF, AFL, and all comorbidities that have been validated or used in prior studies [29,30]. Second, data on medication use and adherence were not available within the NRD. Hence, we were unable to assess data on the specific types or doses of anticoagulants used, or if the patients were prescribed any form of rate or rhythm control. Third, because most patients in both AF and AFL groups were more than 65 years old, our findings may not be generalizable to a younger population. Fourth, as the linkage variable “NRD_VISITLINK” does not carry over across multiple years, the maximum amount of data able to be analyzed for this study is one year’s worth. Therefore, stroke or STE events after one year will not be captured, and as stroke and STE events can take more than one year to develop, our study may not be able to capture the true rate of stroke and STE events in AF and AFL patients. Finally, not excluding patients who underwent ablation procedures is one of the limitations. Maintaining sinus rhythm reduces the risk of stroke. Hence, atrial flutter is more likely to have a lower stroke risk after an ablation procedure. Further prospective cohort studies with longer follow-up and accessible anticoagulation data are needed to understand further and clarify the stroke risk difference between AF and AFL.

## Conclusions

Our evidence suggests there is a decreased risk of stroke and STE events in AFL compared to AF. For AFL, predictors of stroke and STE are similar to those of AF, such as increased age, previous stroke, hypertension, diabetes, and peripheral vascular disease. Studies with anticoagulation information and longer follow-up periods are needed to verify the study results.
